# Regulation of Pollen Tube Growth by Transglutaminase 

**DOI:** 10.3390/plants2010087

**Published:** 2013-03-06

**Authors:** Giampiero Cai, Donatella Serafini-Fracassini, Stefano Del Duca

**Affiliations:** 1 Dipartimento di Scienze della Vita, Università degli Studi di Siena, via Mattioli 4, Siena 53100, Italy; E-Mail: giampiero.cai@unisi.it; 2 Dipartimento di Scienze Biologiche, Geologiche e Ambientali, Università degli Studi di Bologna, via Irnerio, Bologna 40126, Italy; E-Mail: donatella.serafini@unibo.it

**Keywords:** pollen, pollen tube, transglutaminase, cytoskeleton, cell wall, polyamines, protein transport, protein secretion

## Abstract

In pollen tubes, cytoskeleton proteins are involved in many aspects of pollen germination and growth, from the transport of sperm cells to the asymmetrical distribution of organelles to the deposition of cell wall material. These activities are based on the dynamics of the cytoskeleton. Changes to both actin filaments and microtubules are triggered by specific proteins, resulting in different organization levels suitable for the different functions of the cytoskeleton. Transglutaminases are enzymes ubiquitous in all plant organs and cell compartments. They catalyze the post-translational conjugation of polyamines to different protein targets, such as the cytoskeleton. Transglutaminases are suggested to have a general role in the interaction between pollen tubes and the extracellular matrix during fertilization and a specific role during the self-incompatibility response. In such processes, the activity of transglutaminases is enhanced, leading to the formation of cross-linked products (including aggregates of tubulin and actin). Consequently, transglutaminases are suggested to act as regulators of cytoskeleton dynamics. The distribution of transglutaminases in pollen tubes is affected by both membrane dynamics and the cytoskeleton. Transglutaminases are also secreted in the extracellular matrix, where they may take part in the assembly and/or strengthening of the pollen tube cell wall.

## 1. The Pollen and Its Germination

The reproductive success of seed plants is performed and based on the invention of the pollen tube, an extension of the pollen grain that is produced under favorable conditions and that allows the sperm cells to move from the male gametophyte towards the female one. The invention of this biological road made it possible for land plants to reproduce in either the absence or the scarcity of water, thus increasing the reproductive success and dispersion of plants on earth. From a pure biological point of view, the pollen tube is an atypical plant cell, because it grows by a tip-growing mechanism, rather than by the typical diffuse way of somatic cells. The fact that pollen tubes grow exclusively in a specific domain (the tip) following a precise direction suggested that pollen tubes might be compared not only to rhizodermis trichoblastic cells, but also to nerve cells of animals [1]. Such a comparison is clearly forced and may simply stress the similarities between two very different cell types. The pollen tube grows by accumulating secretory vesicles in the tip domain. Vesicles are transported, together with many other organelles, along the cytoskeleton. The driving force is mainly based on the dynamic interaction between actin filaments (AFs) and myosins, although microtubules (MTs) and related motor proteins may have a regulatory function. Vesicles, which are produced by Golgi bodies, contain components required for the assembly of the cell wall and for maintaining the growth process. Two types of cell wall components are delivered to the tip domain: pectins/hemicelluloses and enzymes required for the membrane-localized synthesis of callose and cellulose. Pectins are used to form the primary wall of pollen tubes, a viscoelastic cell wall that allows expansion of the pollen tube by turgor pressure [2]. On the other hand, cellulose and callose are necessary for the strengthening of the cell wall [3], as well as for stabilizing the growth direction of pollen tubes [4]. Although the anatomy of pollen tubes is different from that of somatic cells, the growth of pollen tubes is likely to be based on the balance between the force exerted by turgor pressure and mechanical resistance due to the relative content of esterified/acid pectins. By interchanging the pectin composition of the apical cell wall, pollen tubes can grow by expanding such a wall through the force exerted by turgor pressure. Close to the growth domain, callose and (to a lesser extent) cellulose are deposited by their plasma membrane-localized enzymes [5]. This process reinforces and stabilizes the pollen tube cell wall. These preliminary concepts suggest that the composition of the cell wall is critical to determine the growth rate of pollen tubes. Therefore, every protein that can modify either the structure of the cell wall or the regulation of the growth process is elected as a key factor for the tip-growing mechanism. 

Secretory vesicles are also likely to deliver proteins and other molecules required for regulating the growth process. These include receptors, ion channels, plasma membrane-associated G proteins and lipid-modifying enzymes. This network of proteins is necessary to maintain the molecular interplay that finely regulates growth. Regulation is critical, because pollen tube growth must be synchronized with external signals from the extracellular matrix (ECM) of surrounding cells (the pollen-pistil extracellular matrix—psECM—in the case of the female environment) [6]. It is obvious that the synchronization mechanism is linked to the assembly process of the cell wall (although such a correlation is largely unknown). Currently, accepted models indicate that the balance between pectin composition and turgor pressure constitutes the “motor” that promotes tube growth, while the molecular network in the tip domain represents the “driver” that either guides the growth or regulates its rate. 

## 2. Structure and Function of TGases

Fifty years ago, a transamidating activity was described in liver and attributed to transglutaminases (TGs; EC 2.3.2.13) (TGase) [7]. Thereafter, many reports showed that the enzyme family responsible for this activity is ubiquitous in microorganisms, plants, invertebrates and vertebrates [8,9]. Twenty five years ago, the presence of a TGase activity was in fact reported in higher plants, and its characteristics were reported in some reviews [10,11].

**Figure 1:**
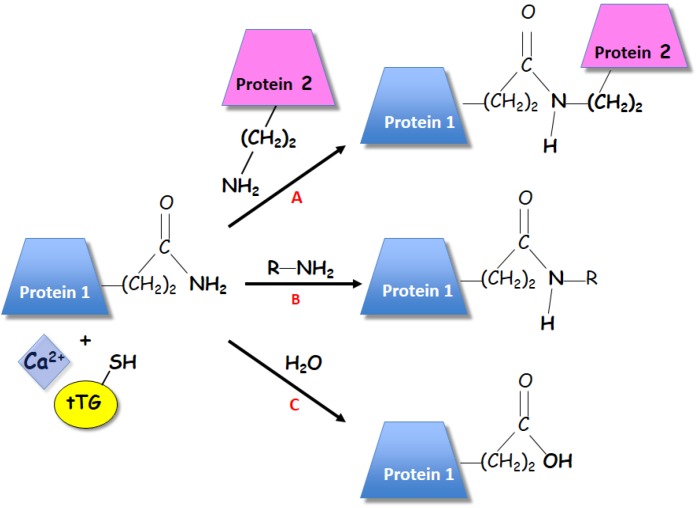
Transglutaminase could catalyze different biochemical reactions. Among them, (**A**) the Ca^2+^-dependent acyl-transfer reaction between the γ-carboxamide group of a specific protein-bound glutamine and the ε-amino group of a distinct protein-bound lysine, residue giving rise to protein crosslinking. Second, the incorporation of substrates having a primary amino group, as polyamines, to the γ-carboxamide group of a specific protein-bound glutamine (**B**). Third, the deamidation of glutamyl-residue to a glutamic acid (**C**). This reaction occurs in the absence of a primary amine.

Since its first description as an enzyme capable of calcium-dependent transamidation (7) (Figure 1), these enzymes were shown to catalyze the post-translational modification of proteins via either transamidation of selected glutamine residues to lysine ones or through covalently binding to primary amines. Because they form stable intra- and inter-molecular bridges, these enzymes are often referred to as “biological glues” [12]. TGases display strict specificity in recognition of glutamine protein substrates; however, the rules, which govern selection of only a few peptidyl glutamine residues, are unclear [13] and have poor specificity for the acyl-acceptor amine group. The transamidation of glutamine residues occurs to a variety of primary amines, which can be the ε-amino group of peptidyl lysine (Figure 1A) or the primary amine of a low molecular mass (Figure 1B) [14], such as aliphatic polyamines (PAs). In the absence of a suitable amine, water can act as an alternative nucleophile, leading to deamidation of the glutamine residue to glutamate (Figure 1C).

**Figure 2:**
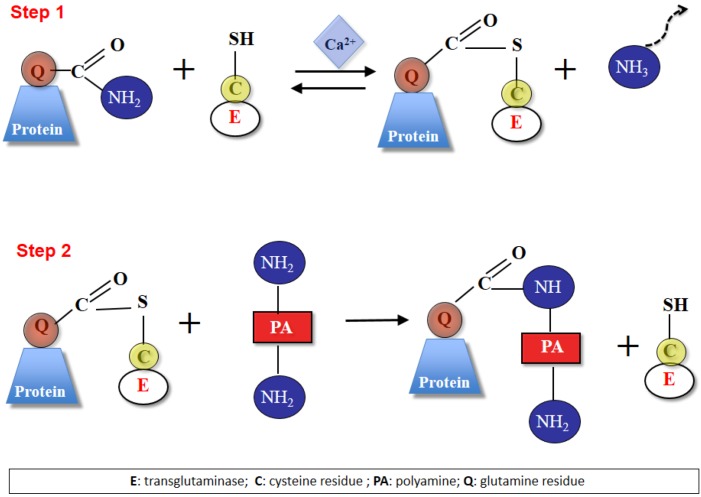
The two-steps transamidase reaction of transglutaminase. All mammalian transglutaminases (TGases) belong to a superfamily of cysteine proteases, have structural homology and possess the catalytic triad of Cys-His-Asp/Asn; the reactivity of this Cys is activated by Ca^2+^, which causes a conformational change in the enzyme, allowing the access of the substrate to the binding site. Step 1: the active Ca^2+^-stabilized conformation of the enzyme forms a covalent intermediate between the active site thiol residue and a glutamyl residue in the protein substrate, releasing ammonia and activating the glutamine acyl moiety. Step 2: the active thioester undergoes an acyl transfer to a primary amine, in this case a polyamine, thus also introducing extra positive charges, as polyamines are protonated at physiological pH [or (not shown) to the lysyl residue of another protein]. A secondary cross-link might form between the free amine group of the bound polyamine and a glutamyl residue in another protein substrate, thus forming bis-(γ-glutamyl)-PA derivatives (not shown). E, Transglutaminase; C, cysteine residue; PA, polyamine; Q, glutamine residue.

The catalytic mechanism is evolutionarily related to that of cysteine proteases. The active site thiol reacts with a glutamine side chain of a protein or peptide substrate to form a thioester intermediate from which the acyl group is transferred to an amine substrate (polyamines in Figure 2). As PAs have two terminal primary amino groups, one or both could be involved in TGase catalysis, leading to the formation of mono-γ-glutamyl or bis-γ-glutamyl derivatives, respectively. As PAs are polycations, these reactions could increase the electric charge of the modified proteins; in the case of bis-γ-glutamyl derivatives, the PA molecule could act as a bridge (with a different length according to the type of PA involved) between endo-glutamyl residues belonging to two different proteins or to the same proteins [7,15]. This catalytic activity, which requires Ca^2+^, is inhibited by guanine nucleotides [16]. In the presence of guanosine triphosphate (GTP), TG2 lacks enzyme activity; instead, it may function as a G protein in the phospholipase C signaling pathway. Whereas the function of TGases is common to all the organisms studied until now, its function as a G protein has not been examined in plants. 

Both reactions involving protein cross-linking and polyamination are relevant *in vivo*, and competition between these amine substrates may take place within cells in a number of important physiological functions. The resulting cross-linked protein structures add strength to tissues and increase their resistance to chemical and proteolytic degradation [12,17].

In animals, nine TGase isoenzymes have been identified at the genomic level, but only six have been isolated and characterized at the protein level. The best-known are the ubiquitous type 2 tissue TGase (tTGase) and plasma Factor XIIIa, involved in the stabilization of fibrin clot. tTGase has been related to an increasing number of other biochemical capacities [9]. This enzyme is located both intra- (cytosol, mitochondria, nucleus) and extra-cellularly in the matrix, where it appears to be involved in differentiation, transmembrane signaling, cell adhesion organization of the extracellular matrix, motility, and pro- and anti-apoptotic roles [18,19]. The structural organization of TGase consists in four domains highly conserved during evolution among the mammalian TGase isoforms [12], as shown by the best known TGase, human type 2 tTGase. 

## 3. Polyamines and Transglutaminases in Plants

Environmental and internal factors regulate plant growth. Among these, PAs are essential rejuvenation growth substances in all living organisms capable of regulating organogenesis and cell proliferation in higher plants and algae, apical growth of pollen, dormancy break, as well as senescence and homeostatic adjustments in response to external stimuli and stresses [20,21]. The first report on PA effects on plant growth was generated by Bertossi *et al.* [22] in *Helianthus tuberosus* dormant tubers and further confirmed in several other plants. The molecular mechanism of action of PAs is only partially known. These amines are present in free and bound form. Twenty-five years ago, interest on the possible role of TGase in plants arose from its known ability to covalently bind PAs to some animal proteins and, thus, possibly also to plant proteins. Despite several characteristics in common with the best known mammalian TGases, among which are the production of glutamyl-PA derivatives and the immunoreactivity by TGase animal antibodies and other characteristics, research on plant TGases has been hampered by difficulties encountered in their purification and in the lack of significant amino acid sequence homologies between animal TGases and the polypeptide sequences available in plant databases. A more recent computational analysis identified in *Arabidopsis thaliana* the presence of a gene, *AtPng1p*, which encodes a putative *N*-glycanase containing the Cys-His-Asp triad typical of the TGase catalytic domain. *AtPng1p* is a single gene expressed ubiquitously, but at low levels, as shown by nested real-time polymerase chain reaction RT-PCR undertaken in different organs of *Arabidopsis*, at all growth stages, in all light assayed conditions [16]. The overexpressed recombinant protein was purified, and an 86-kDa band was immuno-detected. Antibodies raised against this recombinant *AtPng1p* detected the same band in the *Arabidopsis* microsomal fraction and other bands of lower molecular mass in the cytosolic fraction, possibly proteolytic soluble fragments, as usually occur with animal TGases. This finding is in line with the presence of TGase activities and with the immunorecognition of different bands in extracts of several organs of the same plant. Analyses of the γ-glutamyl-derivatives confirmed the *AtPng1p* gene product acts as a TGase, having a Ca^2+^- and GTP-dependent transamidase activity. This was the first plant protein, isolated and characterized at the molecular level, displaying a TGase activity with parameters typically exhibited by animal TGases. A structural comparison between this protein and the crystal of FXIII showed a considerable homology [23]. Despite having an amino acid sequence different from those of known animal TGases, with the exception of the active site triad, *AtPng1p* shares immunological and biochemical properties and possibly an overall similar conformation, probably being a TGase with a different, but convergent, phylogenetic history. The presence of the typical TGase active site in *AtPng1p* elucidates why this protein could exert the same catalytic activity of well-known TGases. AtPng1, which acts as a PNGase when expressed in *Saccharomyces*, possesses two residues for carbohydrate binding that may favor contact among the carbohydrate-PA-protein residues [24]. 

Recently, in *Helianthus tuberosus* immature cells of the primary apical meristem of the sprout, at least three immuno-recognized TGases were found to be expressed and active; these proteins are probably the same already found in differentiated cells of other plant systems, thus representing constitutive enzymes. Two of these TGases (namely the 75 and 85 kD) share a very high degree of molecular mass and amino acid composition similarity with two mammalian TGases, even though no information is available on their amino acid sequence. This suggests that these two TGases are phylogenetically conserved. The third protein of 58 kDa did not show significant similarity with other annotated TGases [25]. Enzymes of such molecular mass are active and distributed in several plants [26]. Two related cDNA, encoding for a putative TGase of 58 kDa, were cloned from *Helianthus* thylakoids; their transcripts were expressed mainly in young leaves and differentiated *Zea mays* callus under light exposure [27]. This TGase did not present the catalytic triad in the position of all annotated TGases and showed no similarity with the 58 kDa of sprout apices [25]. Other protein sequences from various plants can be found in database, where they are classified as TGases only on the basis of their homology with the TGase domain, but they were not experimentally assayed for enzymatic activity. 

The cross-reactivity of the animal TGase antibodies with plant TGases has been reported in several organs and type of plants [28,29]. A similarity between plant and animal TGases is also supported by their catalytic activity, which can cross-recognize some of their respective substrates, suggesting a similarity in their specificity [30,31]. 

In plants, multiple TGase forms are active in the same organ, and many organs/tissues express these enzymes, like, for example, meristems, seeds, flower petals, pollen, leaves, shoots and roots. Most likely, these enzymes are ubiquitous, and some of them may exert similar effects in different cells, as they can occur with the modification of cytoskeleton substrates. On the contrary, other activities appear more specialized in relation to location and substrates, *i.e.*, those typical of plants, like the light-harvesting complexes of chloroplasts [32,33]. 

## 4. Transglutaminase in Pollen

In apple pollen, a Ca^2+^-dependent TGase activity has been found, both intracellularly and extracellularly. TGase is widely distributed in the pollen tube of pear and apple, as well as of hazel (*Corylus avellana*) and pomelo (*Citrus grandis*), and it occurs as both a cytosolic and a membrane/cell wall form [34,35,36,37]. TGase monoclonal and polyclonal antibodies cross-react with a specific polypeptide of ~70 kD in apple pollen, whereas in pear pollen, antibodies cross-reacted with a 70-kD TGase-related polypeptide in the cytoplasmic protein pool, in the membrane (microsomal) fraction and in both Triton- and sodium dodecyl sulfate (SDS)-extracted cell wall proteins. Analyses by two-dimensional gel electrophoresis (2-DE) indicated that each compartment contains distinct isoforms of TGase, suggesting that the pear genome might contain different TGase genes whose products are delivered to different cell regions [33]. Since preliminary observations suggest that the nuclear genomes of *Arabidopsis* and pear contain a single TGase gene [16,34] a single TGase protein seems to be post-translationally modified according to the final destination in the cell. 

In pear [33] and apple pollen [35], TGase was visualized using immunofluorescence and immunogold electron microscopy in the growing half of pollen tubes, suggesting that the enzyme is actively constrained to accumulate in the growing segment. This effect might be possibly achieved by association of TGase with intracellular membranes, as association of gold particles with the plasma membrane of pollen tubes was often observed. This suggests that the enzyme might be either secreted or deposited in order to locally affect the structure of the cell wall, as also confirmed by the immunoblot assay of proteins extracted from different cell compartments. 

### 4.1. Intracellular Targets of TGase Activity: The Cytoskeleton

Starting from initial reports on the binding of putrescine to rabbit skeletal muscle actin mediated by guinea pig liver TGase [38], amines were shown to bind actin covalently by TGase in cells undergoing apoptosis [39]. In addition, G-actin (but not F-actin) was cross-linked with myosin S1 by TGase [40]. Another cytoskeletal target of TGase is myosin [41]. On the tubulin side, MTs were shown to be the target of TGase activity in Alzheimer's disease brains [42]. The MT motor kinesin is also a target of TGase activity [43]. Altogether, these observations suggest that the cytoskeleton activity may be influenced and regulated by TGase activity, either by directly cross-linking specific amino acids or covalently linking PAs to the target surface.

Both AFs and MTs are aligned along the growth axis of the pollen tube and are involved in several aspects of the pollen tube growth mechanism. Generally, AFs are required for the transport of organelles and vesicles in the pollen tube (the so-called “cytoplasmic streaming”) [44]. This process is necessary to deliver Golgi-derived secretory vesicles to the pollen tube apex, where vesicles fuse with the apical plasma membrane and provide new membrane and several components to the developing cell wall [45]. AFs are organized in at least three distinct ways, which are also spatially separated: a delicate meshwork of very short randomly organized AFs in the tip, an actin fringe in the subapex of pollen tubes (that separates the growing domain) and thick actin bundles that permeate the pollen tube to the grain. Bundles are required for the transport of organelles and for their relatively uniform distribution along the pollen tube [46]. The presence of three sub-domains of AFs suggests that an individual regulation of their organization is required at distinct cell locations, a process that definitely requires the presence of specific AF-binding proteins [47], Rac/Rop small GTPases [48] and oscillations of Ca^2+^ concentrations [49]. In summary, AFs are regulated by a complicated network of small molecules and proteins that finely tune AFs in order to promote the coordinated growth of pollen tubes. 

Much less is known about the organization and dynamics of MTs. Immunocytochemical labeling allowed the visualization of cortical MT bundles in the pollen tube of several species [50]. As the use of green fluorescent protein (GFP)-labeled probes for visualizing MTs in living pollen tubes is still not standardized, with a few exceptions [51], the presence and organization of MTs in the apex and subapex of pollen tubes is uncertain. MTs are likely to be involved in the deposition of callose plugs [52], in the transport of sperm cells [53] and in the regulation of organelle/vesicle dynamics [44]. 

The crude extract of apple (*Malus domestica*) pollen contained a TGase activity that catalyzed the incorporation of polyamines into pollen proteins with molecular masses of 43 kD and 52–58 kD; the same proteins were also immunolabeled by mouse monoclonal antibodies to actin and tubulin [54]. TGase purified from apple pollen by hydrophobic interaction chromatography showed molecular and immunological characteristics very similar to those of the well-known TGase-2, which is widely expressed in many animal cells. The pollen enzyme catalyzed the binding of putrescine to actin and tubulin monomers purified from the same cell type. When tested on actin filaments, pollen TGase induces the formation of high-molecular-mass aggregates of actin. Use of fluorescein-cadaverine showed that the labeled polyamine was bound to actin by pollen TGase, as observed to occur with guinea pig liver TGase. The pollen TGase affected the binding of myosin and kinesin to actin filaments and microtubules, respectively [55], and also reduced the enzyme activity of myosin and the gliding activity of kinesin. 

Either excessive concentrations or the absence of PAs disturbed the correct polymerization of actin in the presence of TGase: unordered bundles of actin appeared at high PA concentrations, suggesting that the actin binding sites are possibly saturated, giving rise to many mono-PA derivatives instead of forming the correct site-specific cross-links by bis-PA derivatives [41]. The natural PA concentrations (estimated to be approx. 50 μM in hydrated pollen) should decrease the competition for the protein-binding sites, allowing bis-derivatives to form. Consequently, pollen TGase may participate in actin regulation during pollen tube growth. Since the proper organization of the actin cytoskeleton is essential for tube growth and organelle movement [56], any disorganization of the motor apparatus generates critical changes in organelle motility and pollen tube growth. It was suggested that pollen TGase controls the transition between actin bundles and short filaments at the boundary between the apical and base domains of pollen tubes [54], favoring the assembly of more stable bundles. 

TGase also changes the polymerization rate of tubulin in the presence of PAs. Putrescine at the presumptive natural concentration generated either filamentous or amorphous microtubule structures *in vitro*; motility assays show that TGase-modified microtubules moved with lower speed (at least a 50% reduction) compared with unmodified microtubules. Thus, pollen TGase can act as a “biological glue”, because of its capacity to cross-link proteins and to reduce their movement ability, in agreement with the evidence that the motor activity of kinesin is also regulated by post-translational modification of tubulin [55,57]. The relevance of these findings is demonstrated by the fact that the functioning of pollen tube microtubules involves the control of vacuole positioning, the trafficking velocity of mitochondria and the focusing of secretory vesicles in the tip domain [58]. Since *in vitro* experiments showed that pollen TGase affects the organization and functioning of the cytoskeleton, the authors propose that the activity of TGase might be modulated also *in vivo* to exert a regulatory role on pollen tube growth. 

Recent results suggest that TGase might be involved in the rearrangement of the cytoskeleton as it occurs during self-incompatibility (SI). In *Papaver rhoeas* (poppy), the SI response requires a specific recognition event between stigma and pollen S proteins. This event triggers a cascade of Ca^2+^-dependent signals, which inhibit tip growth, produce critical changes to organelles, cause the depolymerization of AFs (with concurrent formation of actin foci) and determine a caspase-like protease activity [59]. Since the caspase activity also involves depolymerization of MTs [60], the cytoskeleton of pollen tubes is consequently part of the mechanism that regulates the development of the SI response. In Solanaceae, Rosaceae and Plantaginaceae, the SI response is based on the presence of S-RNases, small proteins with RNase activity that are produced by the pistil and are internalized into the pollen tube by either direct absorption or endocytosis [61]. The internalization process might involve the interaction between C2-domain proteins and arabinogalactan-proteins (AGPs), which might in turn bind to S-RNase [62]. Once internalized, S-RNase might degrade the RNA of incompatible pollen in order to prevent fertilization; however, it is likely that other processes, such as changes to Ca^2+^ concentration and modifications of the AF organization, are also involved [63]. Because of this evidence, the molecular mechanisms of rejection of self-pollen might share common features among different families. The molecules that mediate the S-RNase-based SI response are not known, but TGases might be considered a promising candidate. The involvement of TGases in the SI response is suggested by evidence that plant TGases participate to processes correlated to programmed-cell-death (PCD), such as the senescence of the flower corolla [29]. Free PAs declined continuously during the life of the tobacco corolla, but we have shown that the supply of 25 mM spermine for 3 h to flowers at early-mid stages of anthesis delayed senescence and caused diminished fresh weight loss and changes in shape, size and color [64]. Since TGase was immuno-localized in the epidermis and, at senescence, also in the mesophyll cell walls, this suggests the existence of a TGase-mediated mechanism by which PAs modulate senescence and cell death. In addition, the increase of PA content and TGase activity also occurs during incompatible pollinations in pear and citrus [36,65].

One more supporting piece of evidence comes from the finding that the cytoplasmic TGase of apple pollen can post-translationally modify actin and tubulin by conjugating them to PAs [54] and generating high molecular weight aggregates [55]. As mentioned above, such aggregates are capable of inhibiting the enzyme activity and the binding affinity of myosin and kinesin and, consequently, inhibit motor-dependent dynamic activities. The regulatory effect exerted by TGase on MTs may also be indirect; in fact, TGases have stimulatory effects on phospholipase A2 (PLA2) [37] that in turn might regulate the MT dynamics [65,66]. The presence of PLA2 in the pollen of *Arabidopsis thaliana* has been reported only recently [67]. Although plant PLA2s are likely involved in many biological functions (including senescence, wounding and stress responses), relatively little is known about plant PLA2s, and their genes remain essentially uncharacterized. In *Arabidopsis*, three of four PLA2 paralogs (PLA2 β, γ and δ) have been characterized; they are expressed in pollen, are localized in the endoplasmic reticulum and/or Golgi and are critical for pollen development and tube growth [68].

The picture arising from this evidence places TGase at the center of a mechanism that regulates the dynamics of both AFs and MTs under physiological and self-incompatibility conditions. TGase might regulate the dynamics of filamentous cytoskeleton, as well as single associated proteins (motor and non-motor). Such regulation may be direct or even indirect, involving the phospholipase pathways. Dramatic changes to TGase levels, as occurring during the SI response, may consequently lead to the loss of cytoskeleton integrity and function. 

### 4.2. Secretion and Targeting of Extracellular TGase

The pollen tube TGase is also found in association with different membrane compartments, but mainly with Golgi membranes and the plasma membrane [33]. Secretion of TGase in eukaryotic cells might follow alternative routes other than the classic endoplasmic reticulum (ER)/Golgi pathway. This assumption is based on the evidence that TGase is not consistently associated with the Golgi cisternae, suggesting that secretion of extracellular TGase is not dependent on the ER/Golgi pathway [69]. In the pollen tube, TGase accumulates mainly in the subapical region, but not at the apex, where Golgi-derived secretory vesicles are usually present in large amounts [70]. On the other hand, the pollen tube apex is not the only region where secretion occurs [71], and secretion pathways alternative to the ER/Golgi route have been suggested to occur in plant cells [72]. In addition, novel intermediates in the secretory pathway have been identified in plant cells (the so-called “secretory vesicle cluster”) and suggested to be involved in the transport of secretory proteins from the Golgi to the plasma membrane [73]. Consequently, the secretion of TGase might occur in regions out of the tip and involve modalities different from the standard vesicular pathway. 

Association of TGase with the plasma membrane is likely to be dependent on AFs and, to a lesser extent, on MTs, the latter being important for the fine-tuning of membrane insertion [33]). When TGase is bound to the plasma membrane, it is likely to work as a G protein [74], whose GTP binding ability may regulate downstream signaling events involved in cell survival [75]. Since the plasma membrane TGase is likely to activate phospholipase C (PLC) [76], this suggests that the pollen tube TGase associates with PLC and regulate changes of Ca^2+^ concentration through production of inositol 1,4,5-trisphosphate(IP_3_) [77,78].

**Figure 3:**
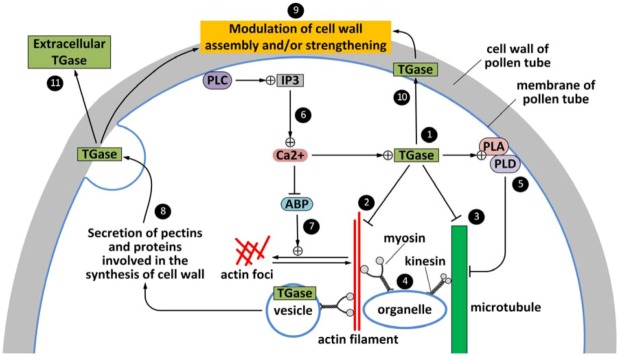
Hypothetical model of TGase trafficking in the pollen tube. Cytoplasmic TGase (**1**) might regulate both actin filaments (**2**) and microtubules (**3**), leading to changes in the cytoskeleton-based organelle transport (**4**). The dynamics of microtubules is also likely regulated by PLA and PLD, the former affected by TGase (**5**). TGase activity may also be controlled through changes of Ca^2+^ concentrations (**6**), which regulate the activity of ABP (**7**). Membrane TGase is secreted either through vesicular intermediates (**8**) or by alternative pathways (**10**), and it might take part in the assembly of the pollen tube cell wall (**9**). TGase may also be secreted in the extracellular matrix (**11**).

The secretion process that should lead to the deposition of TGase in the extracellular matrix is still quite hypothetical, if not speculative. In fact, most of the steps are still not fully clarified, some steps are hypothetical; others, by contrast, are supported by experimental data. The model presented in Figure 3 is, therefore, theoretical, but it also aims to be an incentive for further debate and for future research. Data available in the literature suggests that pollen TGase might be distributed in two distinct locations and be involved in at least two general activities. Cytoplasmic TGase (1) is likely involved in the regulation of cytoskeleton activities by modulating the polymerization state of AFs (2) and MTs (3). This modification may consequently affect the activity of motor proteins (myosin and kinesin) and the motor-based organelle trafficking (4). Regulation of MT dynamics might also be achieved through modulation of phospholipase D (PLD) and A (PLA) activities, the latter enzyme being stimulated by cytoplasmic TGase (5). Cytosolic TGase is supposed to be also controlled by phospholipase C (PLC) through changes of Ca^2+^ concentration (6). Changes of apical Ca^2+^ concentration may affect the activity of actin-binding proteins (ABP), which in turn are likely to control the dynamics of AFs (7) leading eventually to the formation of aberrant structures (actin foci). The second form of TGase is predominantly associated with the membranes and with the cell wall. This form of TGase might be different from the cytosolic, probably being the result of posttranslational modifications. How the membrane TGase can be conveyed within secretory structures is not known. However, membrane-associated TGase is likely to be secreted and incorporated in the cell wall (8) where it participates in its structuring, possibly by interaction with pectins (9). Cell wall-associated TGase may be also the result of an alternative secretory pathway (10). TGase may be also secreted extracellularly (11) and take part in the pollen-pistil signaling interplay (Figure 3).

The presence of extracellular TGase (either incorporated in the cell wall or secreted outside of the cell) is an accepted feature in both animal [12] and plant cells (see below); extracellular TGase is likely to be involved in the stabilization of either the extracellular matrix or the cell wall through its protein cross-linking activity. The first indication of the presence of TGase products in the cell wall was provided by the digestion of cell wall polysaccharides of *Helianthus tuberosus* parenchyma, which caused the disaggregation of PA-conjugated high mass-proteins from polysaccharides [79]. In the alga, *Chlamydomonas reinhardtii*, TGase was involved in the formation of the cell wall, which allowed the zygote to survive desiccation. The TGase-directed formation of a soft protein envelope, which organizes the self-assembly of glycoproteins, was followed by oxidative cross-linking, which rendered the cell wall insoluble. The alga secretes an extracellular 72-kDa TGase, whose maximal activity precedes the insolubilization of the assembled glycoproteins [80]. In the fungus, *Phytophthora sojae*, a cell wall glycoprotein was identified as a Ca^2+^-dependent TGase [81]. In *Nicotiana tabacum* corolla, an active 58-kDa TGase is present in the isolated cell wall fraction and might be responsible for the corolla strengthening finalized to the protection of the ovary containing the developing seeds [29]. The enzyme activity, as measured in isolated cell wall fractions, prevails in the distal part of the corolla and progressively increases during the flower lifespan, with the progression of differentiation and senescence [29]. TGase is immuno-localized almost exclusively in the epidermis, mainly in its cell walls, during corolla development, but at the onset of senescence, the enzyme spreads to the inner parenchymatous cells of the petals, whose cell walls appear rigid and straight, concomitantly with the rigid/papyraceous-like aspect of the corolla [64].

In apple pollen, Western blotting using anti-TGase antibodies also revealed two main immunoreactive bands of 70 and 75 kDa, the latter more evident in crude extracts of germinating pollen, in concentrated germination medium and in the cell wall [35]. 

The presence of TGase in the cell wall has been studied also in pollen by immunolocalization with anti-TGase antibodies and by identification of a TGase cross-linking activity at the apical part of pollen tubes, in the tube region close to the grain and in the pollen grain. The cross-linked products may provide strength to the pollen tube as it migrates through the style *in planta* [82]. As the pollen tip is continuously elongating during pollen germination, released TGase could be deposited along the tube wall and be specifically embedded under the effect of local micro-environmental conditions into the same accumulation sites of the protein cross-links, as shown by immunofluorescence colocalization, both in *in vitro* and *in planta* germinating pollen. The pollen enzyme is also able to catalyze the cross-linking of both mammalian TGase substrates H6-Xpr-GFP (a specific glutamine and lysine rich TGase substrate) and dimethylcasein (the most used substrate for the *in vitro* TGase assay) [35]. In animal tissues, H6-Xpr-GFP revealed a wide distribution of TGase in the extracellular matrices, suggesting an important role for TGase in maintaining the integrity of the extracellular matrix by cross-linking its constituent proteins [83]. 

In pollen, the *in situ* cross-links of H6-Xpr-GFP and pollen proteins were revealed by 81D4, an antibody that recognizes either the gln-lys or gln-PA links and identified by the analysis of the glutamyl-PA derivatives [35]. *In situ* localization by confocal microscopy in non-digested and non-permeabilized pollen revealed that His6-Xpr-GFP was associated extracellularly with the pollen tube wall, especially within 20 μm of the tip. The extracellular pollen TGase co-localized within the same accumulation sites of His6-Xpr-GFP observed along the pollen tube surface. The ability of pollen extracellular TGase to incorporate the exogenously supplied His6-Xpr-GFP in the same sites of pollen protein-protein and protein-amine cross-links was demonstrated by their co-localization in confocal micrographs. In addition, the administration of His6-Xpr-GFP resulted in a significant increase in the percentage of germinated pollen and tube length. The extracellular pollen TGase activity, the percentage of germination and tube growth were inhibited in a dose-dependent manner by the monoclonal antibody ID10 and by two site-directed irreversible inhibitors of TGase activity. The presence of the inhibitors resulted in shorter and thicker pollen tubes with a decreased growth rate within the first period of germination, followed by an inability to extend further the tip and, eventually, tube-burst [35]. The fluorescent-tagged amine substrate (FITC-cadaverine), routinely used to visualize tTGase activity in animal cells [84], which is a competitor of endogenous amine-donor substrates (amino group of lysine residues or PAs), limited or blocked tube elongation of both pollens of *Malus domestica* (entomophilous) and *Corylus avellana* (anemophilous) [37]. This primary amine was essentially detected around the pollen grain of *M. domestica*, mainly in the cell wall and in the apical part of the tube; a similar incorporation of FITC-cadaverine was detected in the pollen tube of *C. avellana* [37].

The identification of the natural substrates of TGase is, thus, of primary importance. Outside the cell of animal tissues, TG2 shapes the extracellular matrix by binding tightly to both fibronectin in the extracellular matrix and integrins on the cell surface; thus, TG2 promotes cell adhesion, signaling and differentiation in a manner independent of its catalytic activity [12]. These proteins can also fulfill other enzymatic activities, according to ligand or substrate availability [9]. In the pollen tube, only a few adhesion molecules have been implicated in guidance [85], and integrin analogues have only been characterized preliminarily in the pollen tube [86]. Whether these proteins are a target of TGase activity is not known. 

In pear pollen, TGase is related to the secretion and/or distribution of methyl-esterified pectins [33]. In fact, methyl-esterified pectins accumulate and are interspersed with respect to the TGase signal. TGase is therefore secreted in the same region and is hypothetically related to the distribution and accumulation of pectins. Results obtained with the inhibitors either of membrane and cytoskeleton mediated-transport, such as Brefeldin A (BFA) oryzalin and Latrunculin B (LatB), indicated that TGase and pectins change their distribution in BFA-treated pollen tubes. Specifically, both TGase and pectins accumulate prominently in older (but not identical) segments of pollen tubes, suggesting that the membrane trafficking inhibitor affects the secretion of both components. On the other hand, LatB induced an apparently different behavior, because TGase accumulates in older segments, but pectins are likely to accumulate in the tip domain. Thus, TGase and pectins follow different secretion pathways that are affected similarly by membrane trafficking inhibitors, but differently by actin inhibitors. While the association of TGase with pectins is an interesting prospective, it is known that PAs can directly interact with polygalacturonic acid or pectins by competing with Ca^2+^ [87]. It can be hypothesized that PAs could be non-covalently bound to pectins by one terminal amino group and covalently linked via TGase to a protein by the other amino terminal group. This binding might regulate the strengthening of pectin-based cell walls and, therefore, the rate of pollen tube growth, which is based on a tug-of-war between turgor pressure and pectin deposition [88]. This hypothesis is also supported by evidence that the TGase AtPng1p possesses two residues for carbohydrate binding that may favor the contact among the carbohydrate-PA-protein residues [24]. TGase also partially overlaps with arabinogalactan proteins (AGPs), complex macromolecules composed of a polypeptide skeleton and branched glycan chains; AGPs are markers of several developmental and interaction processes, such as xylem development, tip growth of pollen tubes, somatic embryogenesis and PCD [89], since AGPs can interact with S-RNase-binding protein during the SI response [90]. AGPs are likely to be secreted at the apex and to be critical in the polarization of new pollen tubes [91]; thus, externalized TGase might interact with AGPs in order to regulate their positioning and/or functioning. These data are consistent with a role of extracellular TGase as the modulator of cell wall building and strengthening. In conclusion, pollen TGase plays an essential role in successful apple pollen tube growth. Therefore, the enzyme could function by protein cross-linking and amine protein conjugation, thus strengthening the cell-wall scaffold of the extending pollen tube.

## 5. Extracellular TGase Released in the Pollen Germination Medium

In apple pollen, two main immunoreactive bands of 70 and 75 kDa were detected in concentrated germination medium in which germinated pollen had been removed by filtration [35]. The extracellular TGase activity is able to conjugate PAs to pollen proteins secreted during germination *in vitro*. *In planta*, it can be hypothesized that the released TGase might be involved in connecting the pollen tube to surrounding stylar cells as it progresses through the psECM [35]. 

By comparing apple and hazel pollen during germination, the pollen grain wall of the entomophilous *M. domestica* was more intensively stained with the anti-TGase antibody than that of the anemophilous *C. avellana* [37]. The enzyme activity increased under stressful treatments, mimicking those induced by climate changes (temperature humidity, copper and acid rain pollution), and was released outside the pollen, along with its products; externalization of TGase was predominant in *C. avellana*, whose grain cell wall is different with respect to that of *M. domestica*. This difference could justify an easier or earlier release of various molecules, including allergens, extracellular TGase and its products by the anemophilous pollens. Pollen TGase could be one of the mediators of pollen allergenicity, especially under environmental stress induced by climate changes. 

Moreover, *M. domestica* and *C. avellana* pollens also differ in their incompatibility mechanisms. *M. domestica* is gametophytic, while *C. avellana* is sporophytic, with the pollen/style recognition times more rapid in hazel, whose incompatibility glycoproteins are exposed to and immediately recognized by stigma cells. 

## 6. Conclusions and Future Perspectives

Recent studies suggest that TGase is a relatively critical element in the growth process of pollen tubes. This activity could be carried out at least at two different levels. The cytoplasmic TGase would be involved, not only as clearly established in the cell free system, but also *in vivo*, in the general organization of the cytoskeletal apparatus that would be made more or less dynamic and, therefore, adapted to the growth rate of pollen tubes. TGase of membranes (or of cell wall) may be somewhat involved in the process of cell wall structuring in order to make it more or less elastic, depending on the medium that the pollen tube has to cross. This possible function is very interesting, because the structure of the cell wall clearly determines the growth speed of pollen tubes, which is also affected by the extracellular TGase acting directly on the cell wall. In fact, the deposition rate of methyl-esterified pectins and their enzymatic transformation in acid pectins is the “timer” that determines how fast the pollen tube can grow. The progressive transformation of pectins counterbalances the turgor pressure inside the pollen tube, setting up a mechanism that is able to respond effectively to changes of the external medium in which the pollen tube grows. Therefore, the membrane/cell wall TGase could be part of the mechanism that makes the cell wall more or less soft. It will therefore be important to check this aspect, determining the role of TGase in counterbalancing the internal force generated by turgor pressure. As the stabilization of the cell wall contributes to determining the growth direction of the pollen tube, the question is whether TGase may have an additional role in this process. In this specific context, the role of TGase would certainly be more important *in vivo* than *in vitro*. 

When the activity of cell wall TGase increases above physiological levels, this may unleash a series of events that would ultimately lead to blocking pollen tube growth. This is the case that might occur during pollen rejection in the SI process. The increase of the enzyme activity of TGase, as triggered by SI signals, may determine, on the one hand, profound changes of the cytoskeleton and, on the other hand, an abnormal process of cell wall restructuring, with consequent damage to the growth process. It will be therefore important to study and analyze the activity and the targets of TGase activity under the SI response, for example, by mimicking *in vitro* the conditions that induce the SI response *in vivo*. 
